# Monomers, dimers, and trimers of [Au(CN)_2_]^−^ in a Ba(diaza-18-crown-6)^2+^ coordination polymer

**DOI:** 10.1107/S1600536809005285

**Published:** 2009-02-21

**Authors:** Christine M. Beavers, Latisha Paw U, Marilyn M. Olmstead

**Affiliations:** a Department of Chemistry, University of California, Davis, CA 95656, USA

## Abstract

The structure of the title compound, poly[triaquatetra-μ-cyanido-tetracyanidobis­(1,4,10,13-tetra­oxa-7,16-diaza­cyclo­octa­deca­ne)di­barium(II)tetra­gold(I)], [Au_4_Ba_2_(CN)_8_(C_12_H_26_N_2_O_4_)_2_(H_2_O)_3_]_*n*_, displays O—H⋯N hydrogen bonding between water molecules and cyano ligands and an unusual pattern of aurophilic inter­actions that yields a monomer, dimer, and trimer of [Au(CN)_2_]^−^ within the same crystal structure. In two of the five Au positions, the atom resides on a center of inversion. The overall arrangement is that of a coordination polymer assisted by aurophilic and hydrogen-bonded inter­actions.

## Related literature

For aurophilic inter­actions, see: Anderson *et al.* (2007 ▶); Schmidbaur (1995 ▶); Pathaneni & Desiraju (1993 ▶). For the structure of a related Pt(CN)_4_^2−^ salt, see: Olmstead *et al.* (2005 ▶).
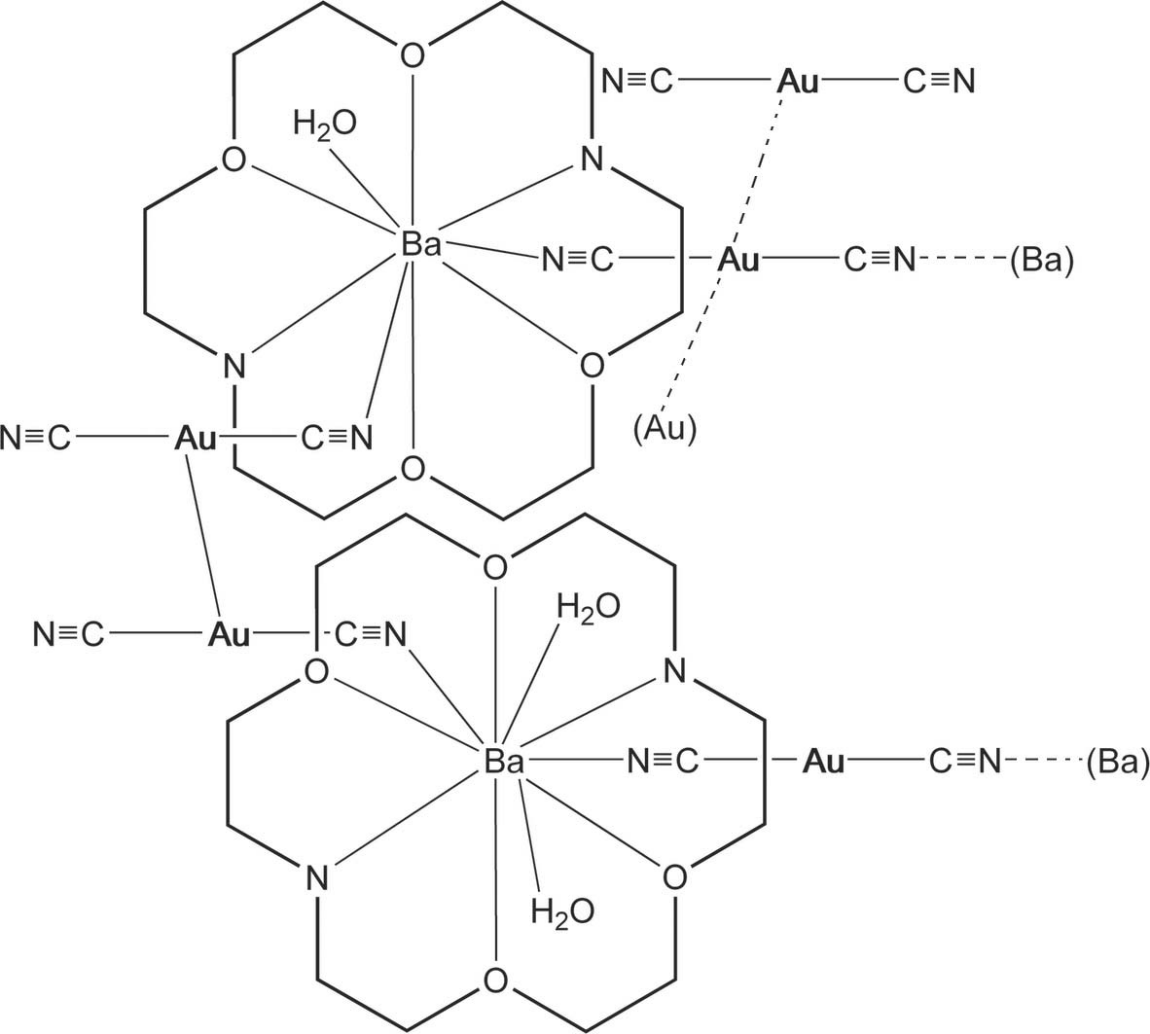


## Experimental

### 

#### Crystal data

[Au_4_Ba_2_(CN)_8_(C_12_H_26_N_2_O_4_)_2_(H_2_O)_3_]
*M* *_r_* = 1849.45
Triclinic, 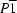

*a* = 11.0962 (3) Å
*b* = 15.9223 (5) Å
*c* = 16.5480 (5) Å
α = 64.142 (2)°
β = 70.523 (2)°
γ = 79.027 (3)°
*V* = 2476.90 (13) Å^3^
*Z* = 2
Mo *K*α radiation
μ = 13.43 mm^−1^
*T* = 90 K
0.20 × 0.15 × 0.12 mm

#### Data collection

Bruker SMART APEXII diffractometer
Absorption correction: multi-scan (*SADABS*; Sheldrick, 1996 ▶) *T*_min_ = 0.174, *T*_max_ = 0.296 (expected range = 0.117–0.200)
46665 measured reflections
15056 independent reflections
13234 reflections with *I* > 2σ(*I*)
*R*_int_ = 0.028

#### Refinement

*R*[*F* ^2^ > 2σ(*F* ^2^)] = 0.021
*wR*(*F* ^2^) = 0.041
*S* = 1.03
15056 reflections
585 parameters
13 restraints
H atoms treated by a mixture of independent and constrained refinement
Δρ_max_ = 1.06 e Å^−3^
Δρ_min_ = −0.89 e Å^−3^

### 

Data collection: *APEX2* (Bruker, 2007 ▶); cell refinement: *SAINT* (Bruker, 2007 ▶); data reduction: *SAINT*; program(s) used to solve structure: *SHELXS97* (Sheldrick, 2008 ▶); program(s) used to refine structure: *SHELXL97* (Sheldrick, 2008 ▶); molecular graphics: *XP* in *SHELXTL* (Sheldrick, 2008 ▶); software used to prepare material for publication: *SHELXL97*.

## Supplementary Material

Crystal structure: contains datablocks I, global. DOI: 10.1107/S1600536809005285/pk2153sup1.cif

Structure factors: contains datablocks I. DOI: 10.1107/S1600536809005285/pk2153Isup2.hkl

Additional supplementary materials:  crystallographic information; 3D view; checkCIF report

## Figures and Tables

**Table:** 

Ba1—N1	2.959 (2)
Ba1—N2	2.919 (2)
Ba1—N5	2.889 (3)
Ba1—N6	2.877 (3)
Ba1—O1	2.854 (2)
Ba1—O2	2.827 (2)
Ba1—O3	2.802 (2)
Ba1—O4	2.850 (2)
Ba1—O5	2.764 (2)
Ba2—N9	2.939 (3)
Ba2—N10	2.867 (3)
Ba2—N11	2.929 (3)
Ba2—N12	2.867 (3)
Ba2—O6	2.888 (2)
Ba2—O7	2.884 (2)
Ba2—O8	2.888 (2)
Ba2—O9	2.929 (2)
Ba2—O10	2.859 (2)
Ba2—O11	2.761 (2)
Au1—Au2	3.5655 (2)
Au1—C13	1.978 (3)
Au1—C14	1.991 (3)
Au2—C15	1.986 (3)
Au3—C16	1.985 (3)
Au3—C17	1.990 (3)
Au3—Au4	3.2670 (2)
Au4—C18	1.988 (3)
Au4—C19	1.989 (3)
Au5—C32	1.985 (3)

**Table:** 

C13—Au1—C14	177.12 (14)
C16—Au3—C17	177.40 (13)
C18—Au4—C19	176.60 (12)

**Table:** 

C14—Au1—Au2—C15	53.73 (13)
C13—Au1—Au2—C15	−127.71 (13)
C14—Au1—Au2—C15^i^	−126.27 (13)
C13—Au1—Au2—C15^i^	52.29 (13)
C16—Au3—Au4—C18	−125.59 (12)
C17—Au3—Au4—C18	54.37 (13)
C16—Au3—Au4—C19	55.81 (12)
C17—Au3—Au4—C19	−124.22 (13)

Symmetry code: (i) 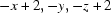.

**Table 2:** Hydrogen-bond geometry (Å, °)

*D*—H⋯*A*	*D*—H	H⋯*A*	*D*⋯*A*	*D*—H⋯*A*
O5—H5*C*⋯N4^i^	0.84 (3)	2.19 (2)	2.997 (4)	161 (4)
O5—H5*D*⋯N3^ii^	0.84 (3)	1.98 (3)	2.804 (4)	168 (3)
O10—H10*C*⋯N8^iii^	0.83 (4)	2.09 (3)	2.916 (3)	170 (3)
O10—H10*D*⋯N6	0.84 (4)	2.35 (2)	3.132 (3)	156 (3)
O11—H11*C*⋯N4^iv^	0.84 (4)	2.01 (3)	2.845 (4)	177 (3)
O11—H11*D*⋯N7^v^	0.84 (3)	2.09 (3)	2.920 (4)	176 (4)

Symmetry codes: (i) 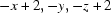; (ii) 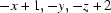; (iii) 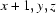; (iv) 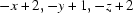; (v) 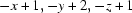.

**Fig. 1.**
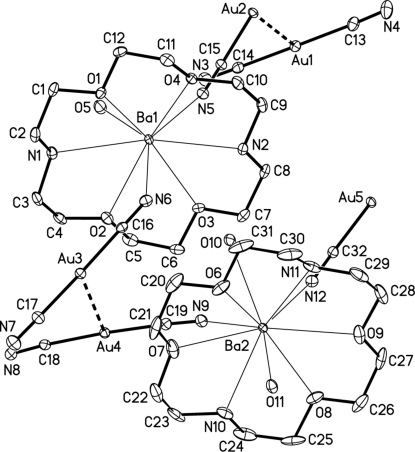
A drawing of the asymmetric unit of the title compound. Thermal ellipsoids are drawn at the 30% probability level. Hydrogen atoms have been omitted for clarity.

**Fig. 2.**
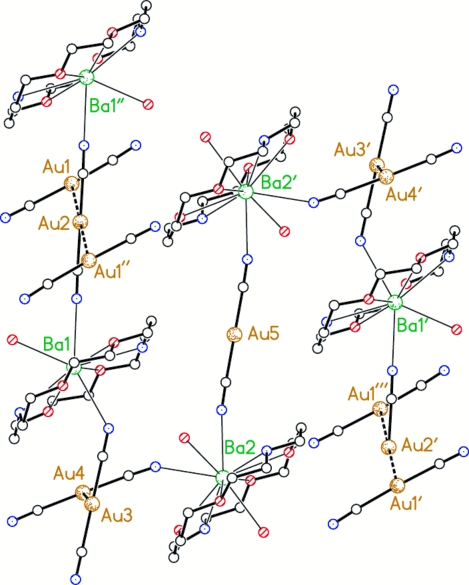
A view that shows how the coordination polymer and the aurophilic interactions are propagated in the title compound. Symmetry codes: (') 2 - x, 1 - y, 2 - z; (") 2 - x, -y, 2 - z; ('") x, 1 + y, z.

**Fig. 3.**
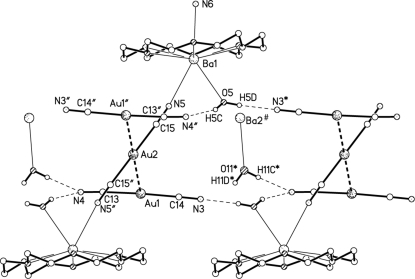
A portion of the hydrogen bonding that occurs between aqua groups and cyanide groups. Symmetry codes: (") 2 - x, -y, 2 - z; (#) 1 - x, -y, 2 - z; (*) 1 - x, 1 - y, 2 - z.

## supplementary crystallographic information

### Comment

Two coordinate gold(I) compounds often associate through aurophilic interactions that span the range of *ca* 2.9 - 3.6 Å (Pathaneni & Desiraju, 1993; Schmidbaur, 1995; Anderson *et al.*, 2007). In previous work (Olmstead *et al.*, 2005) we reported a coordination polymer of Ba(18-crown-6)[Pt(CN)_4_]^.^2H_2_O. The title compound represents an extension of that work to the [Au(CN)_2_]^-^ anion, using a diaza-18-crown-6 to complex Ba^2+^. A related coordination polymer with aurophilic association between gold(I) species is the result.

The asymmetric unit of the title compound consists of two Ba(diaza-18-crown-6)^2+^ cations, four dicyanidoaurate anions, and three molecules of water (Fig. 1). There are five gold positions, two of which, Au2 and Au5, are located on centers of inversion. The monomeric dicyanidoaurate is comprised of Au5, linearly coordinated to two cyanide groups. It functions as a linker anion between two Ba2 complexes *via* N12 of its cyanide group (Fig. 2). It does not participate in any hydrogen bonding nor aurophilic interactions. The closest dicyanidoaurate is that of Au4, at an Au···Au distance of 4.4501 (2) Å. The Au1 and Au2 atoms are involved in the trimer while Au3 and Au4 form the dimer. Distances and angles are reported in Table 1. As shown in Fig. 2, the polymer is connected through a combination of coordination of the [Au(CN)_2_]^-^ nitrogen atoms to barium and aurophilic interactions. All of the hydrogen atoms of the three coordinated waters behave as hydrogen bond donors to N3, N4, N6, N7 and N8 of the cyanide groups (see Table 2). Fig. 3 depicts how a portion of the polymeric structure is supported by these hydrogen bonds.

The bariums, Ba1 and Ba2, have coordination numbers of 9 and 10, respectively. Ba1 is six-coordinated by the diaza-18-crown-6, two [Au(CN)_2_]^-^ anions and one water molecule. It is 0.56 (2) Å out of the N_2_O_4_ plane of the crown, giving *endo* and *exo* faces. One dicyanidoaurate is coordinated to each face while the water molecule coordinates on the *exo* face. The coordination environment of Ba2 is different. Ba2, which is 0.71 (2) Å out of the N_2_O_4_ plane of the crown, is also coordinated by two dicyanidoaurates, but both are found on the *exo* face. Two water molecules are coordinated to Ba2, one on each face. Four of the eight independent cyanide groups are coordinated through their cyanide N atom to a barium (N5, N6, N9, N12). Interestingly, even though the dimer and trimer differ in their Au···Au distances, they show similar C-Au-Au-C torsion angles that are intermediate between eclipsed and staggered. The average value of the two smaller angles is 53° for the trimer and 55° for the dimer (see Table 1 for details).

### Experimental

A salt of Ba[Au(CN)_2_]_2_ was prepared by mixing 162 mg (0.62 mmol) Ba(NO_3_)_2_ and 288 mg (1.0 mmol) K[Au(CN)_2_] in water and heating until both compounds were dissolved. The solution was then put in an ice bath to precipitate out Ba[Au(CN)_2_]_2_. An excess of 1,4,10,13-tetraoxa-7,16-diazacyclooctadecane (diaza-18-crown-6), 400 mg (1.5 mmol) was dissolved in methanol and added to the precipitated material. This solution was placed in a 5 mm diameter glass tube and layered with water. After 24 h, suitable prismatic crystals formed.

### Refinement

Hydrogen atoms on water and aza-N atoms were located in a difference map and subsequently refined with *U*_iso_ = 1.2*U*_eq_(N or O) and distance restraints of 0.84 (1) Å for O—H, 0.88 Å for N—H and H···H of 1.32 (3) Å for water. The C—H geometry was determined by a riding model with idealized geometry and a C—H distance of 0.99 Å. The largest difference map peaks are due to a small amount of conformational disorder in one of the aza crown rings but this was not modeled. The disorder is reflected in somewhat elongated thermal ellipsoids in the cation involving Ba2.

### Crystal data

**Table:**

[Au_4_Ba_2_(CN)_8_(C_12_H_26_N_2_O_4_)_2_(H_2_O)_3_]	*Z* = 2
*M**_r_* = 1849.45	*F*(000) = 1700
Triclinic, *P*1	*D*_x_ = 2.480 Mg m^−^^3^
Hall symbol: -P 1	Mo *K*α radiation, λ = 0.71073 Å
*a* = 11.0962 (3) Å	Cell parameters from 7386 reflections
*b* = 15.9223 (5) Å	θ = 2.4–31.5°
*c* = 16.5480 (5) Å	µ = 13.43 mm^−^^1^
α = 64.142 (2)°	*T* = 90 K
β = 70.523 (2)°	Prism, colorless
γ = 79.027 (3)°	0.20 × 0.15 × 0.12 mm
*V* = 2476.90 (13) Å^3^	

### Data collection

**Table:**

Bruker SMART APEXII diffractometer	15056 independent reflections
Radiation source: fine-focus sealed tube	13234 reflections with *I* > 2σ(*I*)
graphite	*R*_int_ = 0.028
Detector resolution: 8.3 pixels mm^-1^	θ_max_ = 30.5°, θ_min_ = 2.0°
ω scans	*h* = −15→15
Absorption correction: multi-scan (*SADABS*; Sheldrick, 1996)	*k* = −22→22
*T*_min_ = 0.174, *T*_max_ = 0.296	*l* = −23→23
46665 measured reflections	

### Refinement

**Table:**

Refinement on *F*^2^	Primary atom site location: structure-invariant direct methods
Least-squares matrix: full	Secondary atom site location: difference Fourier map
*R*[*F*^2^ > 2σ(*F*^2^)] = 0.021	Hydrogen site location: inferred from neighbouring sites
*wR*(*F*^2^) = 0.041	H atoms treated by a mixture of independent and constrained refinement
*S* = 1.03	*w* = 1/[σ^2^(*F*_o_^2^) + (0.01*P*)^2^ + 2.3943*P*] where *P* = (*F*_o_^2^ + 2*F*_c_^2^)/3
15056 reflections	(Δ/σ)_max_ = 0.004
585 parameters	Δρ_max_ = 1.06 e Å^−^^3^
13 restraints	Δρ_min_ = −0.89 e Å^−^^3^

### Special details

**Table:**

Geometry. All e.s.d.'s (except the e.s.d. in the dihedral angle between two l.s. planes) are estimated using the full covariance matrix. The cell e.s.d.'s are taken into account individually in the estimation of e.s.d.'s in distances, angles and torsion angles; correlations between e.s.d.'s in cell parameters are only used when they are defined by crystal symmetry. An approximate (isotropic) treatment of cell e.s.d.'s is used for estimating e.s.d.'s involving l.s. planes.
Refinement. Refinement of *F*^2^ against ALL reflections. The weighted *R*-factor *wR* and goodness of fit *S* are based on *F*^2^, conventional *R*-factors *R* are based on *F*, with *F* set to zero for negative *F*^2^. The threshold expression of *F*^2^ > σ(*F*^2^) is used only for calculating *R*-factors(gt) *etc*. and is not relevant to the choice of reflections for refinement. *R*-factors based on *F*^2^ are statistically about twice as large as those based on *F*, and *R*- factors based on ALL data will be even larger.

### Fractional atomic coordinates and isotropic or equivalent isotropic displacement parameters (Å^2^)

**Table:**

	*x*	*y*	*z*	*U*_iso_*/*U*_eq_	
Ba1	0.755709 (15)	0.334179 (12)	0.759393 (11)	0.01240 (3)	
Ba2	0.742307 (15)	0.821847 (11)	0.765305 (11)	0.01231 (3)	
Au1	0.836864 (11)	−0.115019 (9)	1.242169 (9)	0.02120 (3)	
Au2	1.0000	0.0000	1.0000	0.01559 (3)	
Au3	0.551751 (11)	0.686535 (8)	0.561101 (8)	0.01723 (3)	
Au4	0.337214 (11)	0.634372 (8)	0.764727 (8)	0.01702 (3)	
Au5	1.0000	0.5000	1.0000	0.01710 (3)	
O1	0.8799 (2)	0.37038 (15)	0.56699 (14)	0.0192 (4)	
O2	0.49357 (19)	0.39306 (16)	0.79701 (15)	0.0212 (4)	
O3	0.6317 (2)	0.37402 (15)	0.91746 (15)	0.0196 (4)	
O4	1.02624 (19)	0.34617 (15)	0.68338 (14)	0.0178 (4)	
O5	0.6461 (2)	0.17261 (17)	0.80175 (19)	0.0304 (6)	
H5C	0.711 (2)	0.141 (2)	0.784 (3)	0.036*	
H5D	0.585 (2)	0.160 (3)	0.791 (3)	0.036*	
O6	0.8761 (3)	0.88170 (17)	0.56832 (16)	0.0346 (6)	
O7	0.6071 (3)	0.88026 (18)	0.62624 (19)	0.0385 (6)	
O8	0.6167 (2)	0.86170 (17)	0.92912 (16)	0.0312 (6)	
O9	0.8862 (3)	0.86207 (18)	0.86293 (18)	0.0361 (6)	
O10	0.8497 (2)	0.67323 (16)	0.70570 (17)	0.0226 (5)	
H10C	0.9171 (19)	0.644 (2)	0.717 (2)	0.027*	
H10D	0.803 (3)	0.6342 (18)	0.711 (3)	0.027*	
O11	0.7417 (2)	1.01450 (16)	0.68894 (17)	0.0241 (5)	
H11C	0.792 (3)	1.042 (2)	0.696 (2)	0.029*	
H11D	0.711 (3)	1.0566 (17)	0.6479 (19)	0.029*	
N1	0.6063 (3)	0.36039 (19)	0.63095 (18)	0.0200 (5)	
H1	0.597 (4)	0.3021 (10)	0.644 (3)	0.032 (11)*	
N2	0.9045 (2)	0.39262 (18)	0.84017 (18)	0.0181 (5)	
H2	0.917 (3)	0.4529 (9)	0.812 (2)	0.030 (10)*	
N3	0.5818 (3)	−0.1406 (2)	1.2162 (2)	0.0325 (7)	
N4	1.0905 (3)	−0.1036 (3)	1.2788 (2)	0.0372 (8)	
N5	0.8290 (2)	0.17401 (19)	0.90908 (18)	0.0203 (5)	
N6	0.7368 (3)	0.53453 (19)	0.66500 (19)	0.0221 (5)	
N7	0.3765 (3)	0.8392 (2)	0.4486 (2)	0.0298 (6)	
N8	0.1005 (3)	0.5907 (2)	0.72896 (19)	0.0249 (6)	
N9	0.5559 (3)	0.68713 (19)	0.81265 (18)	0.0216 (5)	
N10	0.4887 (2)	0.9078 (2)	0.7923 (2)	0.0276 (6)	
H10	0.508 (3)	0.9636 (12)	0.779 (3)	0.033*	
N11	1.0211 (3)	0.8329 (2)	0.6984 (2)	0.0293 (7)	
H11	1.036 (4)	0.7737 (9)	0.712 (3)	0.035*	
N12	0.8064 (3)	0.65633 (19)	0.91243 (18)	0.0217 (5)	
C1	0.8104 (3)	0.3508 (2)	0.5183 (2)	0.0245 (7)	
H1D	0.8024	0.2826	0.5430	0.029*	
H1B	0.8566	0.3734	0.4507	0.029*	
C2	0.6811 (3)	0.3991 (2)	0.5322 (2)	0.0250 (7)	
H1C	0.6900	0.4666	0.5112	0.030*	
H2B	0.6354	0.3918	0.4936	0.030*	
C3	0.4815 (3)	0.4099 (2)	0.6491 (2)	0.0252 (7)	
H3A	0.4272	0.3997	0.6176	0.030*	
H3B	0.4932	0.4778	0.6226	0.030*	
C4	0.4152 (3)	0.3767 (2)	0.7524 (2)	0.0253 (7)	
H4A	0.3309	0.4108	0.7631	0.030*	
H4B	0.4009	0.3091	0.7792	0.030*	
C5	0.4311 (3)	0.3700 (2)	0.8944 (2)	0.0259 (7)	
H5A	0.4316	0.3013	0.9295	0.031*	
H5B	0.3410	0.3949	0.9032	0.031*	
C6	0.5012 (3)	0.4122 (2)	0.9303 (2)	0.0252 (7)	
H6A	0.4999	0.4810	0.8957	0.030*	
H6B	0.4590	0.3976	0.9976	0.030*	
C7	0.7032 (3)	0.4129 (2)	0.9502 (2)	0.0252 (7)	
H7A	0.6608	0.4012	1.0169	0.030*	
H7B	0.7071	0.4812	0.9134	0.030*	
C8	0.8359 (3)	0.3674 (2)	0.9393 (2)	0.0231 (7)	
H8A	0.8832	0.3878	0.9683	0.028*	
H8B	0.8309	0.2987	0.9719	0.028*	
C9	1.0344 (3)	0.3478 (2)	0.8259 (2)	0.0236 (7)	
H9B	1.0292	0.2790	0.8581	0.028*	
H9C	1.0849	0.3674	0.8534	0.028*	
C10	1.1001 (3)	0.3746 (2)	0.7230 (2)	0.0240 (7)	
H10A	1.1085	0.4431	0.6909	0.029*	
H10B	1.1870	0.3438	0.7142	0.029*	
C11	1.0825 (3)	0.3751 (2)	0.5846 (2)	0.0243 (7)	
H11A	1.1733	0.3520	0.5717	0.029*	
H11B	1.0793	0.4442	0.5535	0.029*	
C12	1.0113 (3)	0.3369 (2)	0.5466 (2)	0.0226 (6)	
H12A	1.0491	0.3576	0.4780	0.027*	
H12B	1.0173	0.2678	0.5757	0.027*	
C13	0.9970 (3)	−0.1053 (2)	1.2646 (2)	0.0248 (7)	
C14	0.6740 (3)	−0.1307 (2)	1.2255 (2)	0.0243 (7)	
C15	0.8885 (3)	0.1093 (2)	0.9446 (2)	0.0175 (6)	
C16	0.6695 (3)	0.5908 (2)	0.6263 (2)	0.0179 (6)	
C17	0.4399 (3)	0.7836 (2)	0.4903 (2)	0.0217 (6)	
C18	0.1896 (3)	0.6048 (2)	0.7407 (2)	0.0204 (6)	
C19	0.4775 (3)	0.6657 (2)	0.7950 (2)	0.0188 (6)	
C20	0.8099 (5)	0.8607 (3)	0.5204 (3)	0.0476 (12)	
H20A	0.7989	0.7926	0.5479	0.057*	
H20B	0.8590	0.8799	0.4535	0.057*	
C21	0.6840 (5)	0.9118 (3)	0.5291 (3)	0.0479 (12)	
H21A	0.6960	0.9796	0.5038	0.058*	
H21B	0.6385	0.9021	0.4918	0.058*	
C22	0.4890 (4)	0.9331 (3)	0.6355 (3)	0.0411 (10)	
H22A	0.4389	0.9243	0.6004	0.049*	
H22B	0.5052	1.0003	0.6085	0.049*	
C23	0.4141 (3)	0.9032 (3)	0.7363 (3)	0.0441 (11)	
H23A	0.3352	0.9441	0.7419	0.053*	
H23B	0.3885	0.8384	0.7609	0.053*	
C24	0.4193 (4)	0.8799 (3)	0.8896 (3)	0.0449 (11)	
H24A	0.4107	0.8115	0.9194	0.054*	
H24B	0.3324	0.9107	0.8958	0.054*	
C25	0.4878 (4)	0.9064 (3)	0.9372 (3)	0.0449 (12)	
H25A	0.4934	0.9752	0.9087	0.054*	
H25B	0.4395	0.8873	1.0041	0.054*	
C26	0.6879 (5)	0.8926 (3)	0.9667 (3)	0.0441 (11)	
H26A	0.6419	0.8806	1.0329	0.053*	
H26B	0.6984	0.9607	0.9314	0.053*	
C27	0.8161 (4)	0.8417 (3)	0.9603 (3)	0.0430 (11)	
H27A	0.8651	0.8609	0.9891	0.052*	
H27B	0.8053	0.7736	0.9951	0.052*	
C28	1.0160 (4)	0.8261 (3)	0.8502 (3)	0.0439 (11)	
H28A	1.0189	0.7569	0.8789	0.053*	
H28B	1.0592	0.8475	0.8808	0.053*	
C29	1.0828 (3)	0.8602 (3)	0.7471 (3)	0.0453 (11)	
H29A	1.0841	0.9292	0.7200	0.054*	
H29B	1.1725	0.8346	0.7378	0.054*	
C30	1.0738 (4)	0.8775 (3)	0.5983 (3)	0.0507 (13)	
H30A	1.1665	0.8604	0.5811	0.061*	
H30B	1.0632	0.9462	0.5781	0.061*	
C31	1.0091 (4)	0.8489 (3)	0.5484 (3)	0.0485 (12)	
H31A	1.0509	0.8766	0.4801	0.058*	
H31B	1.0157	0.7801	0.5701	0.058*	
C32	0.8760 (3)	0.5980 (2)	0.9448 (2)	0.0186 (6)	

### Atomic displacement parameters (Å^2^)

**Table:**

	*U*^11^	*U*^22^	*U*^33^	*U*^12^	*U*^13^	*U*^23^
Ba1	0.01125 (7)	0.01328 (8)	0.01126 (7)	−0.00179 (6)	−0.00387 (6)	−0.00274 (6)
Ba2	0.01208 (7)	0.01168 (8)	0.01154 (7)	−0.00047 (6)	−0.00365 (6)	−0.00308 (6)
Au1	0.02148 (6)	0.02113 (6)	0.02549 (6)	0.00010 (5)	−0.00862 (5)	−0.01244 (5)
Au2	0.01522 (7)	0.01358 (7)	0.01744 (7)	0.00157 (6)	−0.00750 (6)	−0.00462 (6)
Au3	0.01753 (5)	0.01549 (6)	0.01742 (5)	−0.00105 (4)	−0.00516 (4)	−0.00528 (4)
Au4	0.01769 (5)	0.01667 (6)	0.01733 (5)	−0.00038 (4)	−0.00625 (4)	−0.00666 (4)
Au5	0.01969 (8)	0.01373 (8)	0.01890 (8)	0.00225 (6)	−0.01073 (6)	−0.00489 (6)
O1	0.0210 (11)	0.0224 (12)	0.0160 (10)	−0.0025 (9)	−0.0038 (8)	−0.0099 (9)
O2	0.0146 (10)	0.0233 (12)	0.0211 (11)	−0.0044 (9)	−0.0029 (8)	−0.0050 (9)
O3	0.0197 (11)	0.0173 (11)	0.0204 (11)	0.0014 (8)	−0.0026 (9)	−0.0096 (9)
O4	0.0138 (10)	0.0186 (11)	0.0186 (10)	−0.0028 (8)	−0.0028 (8)	−0.0057 (9)
O5	0.0321 (14)	0.0211 (13)	0.0455 (16)	0.0010 (11)	−0.0217 (12)	−0.0133 (12)
O6	0.0570 (17)	0.0228 (13)	0.0167 (12)	−0.0110 (12)	0.0039 (11)	−0.0085 (10)
O7	0.0507 (17)	0.0250 (14)	0.0415 (16)	−0.0065 (12)	−0.0318 (14)	0.0006 (12)
O8	0.0482 (16)	0.0201 (12)	0.0181 (12)	0.0000 (11)	−0.0005 (11)	−0.0083 (10)
O9	0.0453 (16)	0.0313 (15)	0.0348 (14)	−0.0091 (12)	−0.0259 (13)	−0.0028 (12)
O10	0.0205 (11)	0.0212 (12)	0.0301 (12)	−0.0005 (9)	−0.0100 (10)	−0.0121 (10)
O11	0.0274 (12)	0.0167 (12)	0.0290 (13)	−0.0023 (9)	−0.0157 (10)	−0.0038 (10)
N1	0.0242 (13)	0.0149 (13)	0.0211 (13)	−0.0020 (11)	−0.0116 (11)	−0.0033 (11)
N2	0.0242 (13)	0.0143 (13)	0.0168 (12)	−0.0027 (10)	−0.0090 (10)	−0.0041 (10)
N3	0.0294 (16)	0.0315 (17)	0.0419 (19)	0.0025 (13)	−0.0157 (14)	−0.0171 (15)
N4	0.0334 (17)	0.053 (2)	0.0303 (17)	−0.0164 (15)	−0.0080 (14)	−0.0169 (16)
N5	0.0198 (13)	0.0196 (14)	0.0206 (13)	−0.0001 (10)	−0.0064 (11)	−0.0071 (11)
N6	0.0242 (14)	0.0173 (13)	0.0232 (14)	−0.0034 (11)	−0.0046 (11)	−0.0075 (11)
N7	0.0289 (15)	0.0247 (16)	0.0292 (16)	−0.0028 (12)	−0.0103 (13)	−0.0029 (13)
N8	0.0240 (14)	0.0292 (16)	0.0251 (14)	0.0018 (12)	−0.0080 (12)	−0.0145 (13)
N9	0.0240 (13)	0.0176 (13)	0.0198 (13)	−0.0013 (11)	−0.0081 (11)	−0.0033 (11)
N10	0.0115 (12)	0.0229 (15)	0.0368 (17)	−0.0031 (11)	0.0001 (11)	−0.0059 (13)
N11	0.0139 (13)	0.0155 (14)	0.0452 (18)	−0.0019 (11)	−0.0018 (12)	−0.0047 (13)
N12	0.0233 (13)	0.0185 (14)	0.0213 (13)	−0.0015 (11)	−0.0060 (11)	−0.0062 (11)
C1	0.0352 (18)	0.0253 (17)	0.0186 (15)	−0.0042 (14)	−0.0113 (14)	−0.0100 (13)
C2	0.0350 (18)	0.0220 (17)	0.0221 (16)	−0.0047 (14)	−0.0163 (14)	−0.0053 (13)
C3	0.0243 (16)	0.0192 (16)	0.0372 (19)	0.0004 (13)	−0.0207 (15)	−0.0078 (14)
C4	0.0152 (14)	0.0203 (16)	0.040 (2)	0.0004 (12)	−0.0115 (14)	−0.0100 (15)
C5	0.0182 (15)	0.0244 (17)	0.0198 (16)	−0.0035 (13)	0.0032 (12)	−0.0006 (13)
C6	0.0243 (16)	0.0229 (17)	0.0210 (16)	0.0068 (13)	−0.0030 (13)	−0.0079 (14)
C7	0.0357 (18)	0.0237 (17)	0.0198 (16)	−0.0030 (14)	−0.0058 (14)	−0.0128 (14)
C8	0.0327 (17)	0.0239 (17)	0.0176 (15)	−0.0064 (14)	−0.0091 (13)	−0.0094 (13)
C9	0.0222 (16)	0.0237 (17)	0.0256 (17)	−0.0095 (13)	−0.0098 (13)	−0.0049 (14)
C10	0.0152 (14)	0.0251 (17)	0.0313 (18)	−0.0070 (12)	−0.0064 (13)	−0.0087 (14)
C11	0.0153 (14)	0.0285 (18)	0.0197 (16)	−0.0054 (13)	0.0036 (12)	−0.0058 (14)
C12	0.0253 (16)	0.0215 (16)	0.0177 (15)	0.0008 (13)	−0.0003 (12)	−0.0099 (13)
C13	0.0278 (17)	0.0277 (18)	0.0198 (16)	−0.0079 (14)	−0.0042 (13)	−0.0096 (14)
C14	0.0296 (17)	0.0190 (16)	0.0306 (18)	0.0036 (13)	−0.0137 (14)	−0.0139 (14)
C15	0.0165 (14)	0.0162 (15)	0.0181 (14)	−0.0011 (11)	−0.0050 (11)	−0.0052 (12)
C16	0.0195 (14)	0.0155 (15)	0.0189 (14)	−0.0038 (11)	−0.0024 (12)	−0.0081 (12)
C17	0.0187 (15)	0.0221 (16)	0.0202 (15)	−0.0026 (12)	−0.0040 (12)	−0.0054 (13)
C18	0.0217 (15)	0.0193 (16)	0.0214 (15)	0.0036 (12)	−0.0083 (12)	−0.0095 (13)
C19	0.0228 (15)	0.0173 (15)	0.0136 (14)	−0.0019 (12)	−0.0051 (12)	−0.0035 (12)
C20	0.095 (4)	0.029 (2)	0.0180 (18)	−0.012 (2)	−0.013 (2)	−0.0068 (16)
C21	0.102 (4)	0.027 (2)	0.0231 (19)	−0.023 (2)	−0.035 (2)	0.0036 (16)
C22	0.048 (2)	0.0212 (19)	0.061 (3)	−0.0042 (17)	−0.041 (2)	−0.0035 (18)
C23	0.0218 (18)	0.0214 (19)	0.096 (4)	0.0069 (15)	−0.032 (2)	−0.022 (2)
C24	0.0221 (18)	0.031 (2)	0.049 (3)	−0.0013 (16)	0.0132 (17)	−0.0044 (19)
C25	0.052 (3)	0.0237 (19)	0.0247 (19)	0.0129 (18)	0.0159 (17)	−0.0049 (16)
C26	0.090 (3)	0.025 (2)	0.0240 (19)	−0.003 (2)	−0.021 (2)	−0.0123 (16)
C27	0.085 (3)	0.029 (2)	0.0265 (19)	−0.019 (2)	−0.034 (2)	−0.0020 (16)
C28	0.045 (2)	0.025 (2)	0.068 (3)	−0.0060 (17)	−0.042 (2)	−0.004 (2)
C29	0.0190 (17)	0.026 (2)	0.090 (4)	0.0009 (15)	−0.021 (2)	−0.020 (2)
C30	0.029 (2)	0.033 (2)	0.071 (3)	−0.0104 (17)	0.023 (2)	−0.027 (2)
C31	0.057 (3)	0.029 (2)	0.035 (2)	−0.0129 (19)	0.0285 (19)	−0.0169 (18)
C32	0.0224 (15)	0.0163 (15)	0.0167 (14)	−0.0014 (12)	−0.0069 (12)	−0.0051 (12)

### Geometric parameters (Å, °)

**Table:**

Ba1—N1	2.959 (2)	N10—C23	1.463 (5)
Ba1—N2	2.919 (2)	N10—H10	0.87 (3)
Ba1—N5	2.889 (3)	N11—C30	1.441 (5)
Ba1—N6	2.877 (3)	N11—C29	1.447 (5)
Ba1—O1	2.854 (2)	N11—H11	0.87 (3)
Ba1—O2	2.827 (2)	N12—C32	1.147 (4)
Ba1—O3	2.802 (2)	C1—C2	1.489 (5)
Ba1—O4	2.850 (2)	C1—H1D	0.9900
Ba1—O5	2.764 (2)	C1—H1B	0.9900
Ba2—N9	2.939 (3)	C2—H1C	0.9900
Ba2—N10	2.867 (3)	C2—H2B	0.9900
Ba2—N11	2.929 (3)	C3—C4	1.505 (5)
Ba2—N12	2.867 (3)	C3—H3A	0.9900
Ba2—O6	2.888 (2)	C3—H3B	0.9900
Ba2—O7	2.884 (2)	C4—H4A	0.9900
Ba2—O8	2.888 (2)	C4—H4B	0.9900
Ba2—O9	2.929 (2)	C5—C6	1.505 (5)
Ba2—O10	2.859 (2)	C5—H5A	0.9900
Ba2—O11	2.761 (2)	C5—H5B	0.9900
Au1—Au2	3.5655 (2)	C6—H6A	0.9900
Au1—C13	1.978 (3)	C6—H6B	0.9900
Au1—C14	1.991 (3)	C7—C8	1.502 (5)
Au2—C15^i^	1.986 (3)	C7—H7A	0.9900
Au2—C15	1.986 (3)	C7—H7B	0.9900
Au3—C16	1.985 (3)	C8—H8A	0.9900
Au3—C17	1.990 (3)	C8—H8B	0.9900
Au3—Au4	3.2670 (2)	C9—C10	1.506 (4)
Au4—C18	1.988 (3)	C9—H9B	0.9900
Au4—C19	1.989 (3)	C9—H9C	0.9900
Au5—C32^ii^	1.985 (3)	C10—H10A	0.9900
Au5—C32	1.985 (3)	C10—H10B	0.9900
O1—C12	1.434 (4)	C11—C12	1.495 (4)
O1—C1	1.438 (3)	C11—H11A	0.9900
O2—C5	1.432 (4)	C11—H11B	0.9900
O2—C4	1.432 (4)	C12—H12A	0.9900
O3—C7	1.440 (4)	C12—H12B	0.9900
O3—C6	1.443 (4)	C20—C21	1.474 (6)
O4—C11	1.431 (4)	C20—H20A	0.9900
O4—C10	1.437 (3)	C20—H20B	0.9900
O5—H5C	0.84 (3)	C21—H21A	0.9900
O5—H5D	0.83 (3)	C21—H21B	0.9900
O6—C20	1.413 (5)	C22—C23	1.494 (6)
O6—C31	1.447 (5)	C22—H22A	0.9900
O7—C22	1.417 (5)	C22—H22B	0.9900
O7—C21	1.452 (5)	C23—H23A	0.9900
O8—C26	1.412 (5)	C23—H23B	0.9900
O8—C25	1.462 (5)	C24—C25	1.478 (6)
O9—C28	1.427 (5)	C24—H24A	0.9900
O9—C27	1.453 (5)	C24—H24B	0.9900
O10—H10C	0.83 (4)	C25—H25A	0.9900
O10—H10D	0.84 (4)	C25—H25B	0.9900
O11—H11C	0.84 (4)	C26—C27	1.492 (6)
O11—H11D	0.84 (3)	C26—H26A	0.9900
N1—C3	1.464 (4)	C26—H26B	0.9900
N1—C2	1.467 (4)	C27—H27A	0.9900
N1—H1	0.88 (3)	C27—H27B	0.9900
N2—C8	1.463 (4)	C28—C29	1.501 (6)
N2—C9	1.473 (4)	C28—H28A	0.9900
N2—H2	0.88 (3)	C28—H28B	0.9900
N3—C14	1.134 (4)	C29—H29A	0.9900
N4—C13	1.145 (4)	C29—H29B	0.9900
N5—C15	1.147 (4)	C30—C31	1.500 (6)
N6—C16	1.155 (4)	C30—H30A	0.9900
N7—C17	1.145 (4)	C30—H30B	0.9900
N8—C18	1.147 (4)	C31—H31A	0.9900
N9—C19	1.152 (4)	C31—H31B	0.9900
N10—C24	1.437 (5)		
			
O5—Ba1—O3	102.83 (7)	O1—C1—H1D	110.0
O5—Ba1—O2	79.48 (7)	C2—C1—H1D	110.0
O3—Ba1—O2	59.26 (6)	O1—C1—H1B	110.0
O5—Ba1—O4	119.33 (7)	C2—C1—H1B	110.0
O3—Ba1—O4	119.43 (6)	H1D—C1—H1B	108.4
O2—Ba1—O4	159.10 (6)	N1—C2—C1	111.0 (3)
O5—Ba1—O1	96.10 (7)	N1—C2—H1C	109.4
O3—Ba1—O1	157.80 (6)	C1—C2—H1C	109.4
O2—Ba1—O1	114.11 (6)	N1—C2—H2B	109.4
O4—Ba1—O1	58.19 (6)	C1—C2—H2B	109.4
O5—Ba1—N6	143.22 (7)	H1C—C2—H2B	108.0
O3—Ba1—N6	82.11 (7)	N1—C3—C4	111.3 (3)
O2—Ba1—N6	71.92 (7)	N1—C3—H3A	109.4
O4—Ba1—N6	87.18 (7)	C4—C3—H3A	109.4
O1—Ba1—N6	75.77 (7)	N1—C3—H3B	109.4
O5—Ba1—N5	66.95 (7)	C4—C3—H3B	109.4
O3—Ba1—N5	76.74 (7)	H3A—C3—H3B	108.0
O2—Ba1—N5	115.92 (7)	O2—C4—C3	108.9 (2)
O4—Ba1—N5	81.94 (7)	O2—C4—H4A	109.9
O1—Ba1—N5	122.06 (7)	C3—C4—H4A	109.9
N6—Ba1—N5	147.29 (7)	O2—C4—H4B	109.9
O5—Ba1—N2	136.59 (7)	C3—C4—H4B	109.9
O3—Ba1—N2	60.51 (7)	H4A—C4—H4B	108.3
O2—Ba1—N2	114.90 (7)	O2—C5—C6	108.6 (3)
O4—Ba1—N2	58.94 (7)	O2—C5—H5A	110.0
O1—Ba1—N2	111.94 (7)	C6—C5—H5A	110.0
N6—Ba1—N2	77.83 (7)	O2—C5—H5B	110.0
N5—Ba1—N2	70.12 (7)	C6—C5—H5B	110.0
O5—Ba1—N1	67.30 (7)	H5A—C5—H5B	108.3
O3—Ba1—N1	118.09 (7)	O3—C6—C5	108.8 (3)
O2—Ba1—N1	58.85 (7)	O3—C6—H6A	109.9
O4—Ba1—N1	117.43 (7)	C5—C6—H6A	109.9
O1—Ba1—N1	59.24 (7)	O3—C6—H6B	109.9
N6—Ba1—N1	78.16 (7)	C5—C6—H6B	109.9
N5—Ba1—N1	134.01 (7)	H6A—C6—H6B	108.3
N2—Ba1—N1	155.87 (7)	O3—C7—C8	108.5 (2)
O11—Ba2—O10	137.70 (7)	O3—C7—H7A	110.0
O11—Ba2—N12	143.24 (7)	C8—C7—H7A	110.0
O10—Ba2—N12	67.64 (7)	O3—C7—H7B	110.0
O11—Ba2—N10	67.46 (8)	C8—C7—H7B	110.0
O10—Ba2—N10	131.20 (8)	H7A—C7—H7B	108.4
N12—Ba2—N10	120.42 (8)	N2—C8—C7	110.5 (3)
O11—Ba2—O7	75.90 (7)	N2—C8—H8A	109.5
O10—Ba2—O7	84.97 (7)	C7—C8—H8A	109.5
N12—Ba2—O7	140.41 (7)	N2—C8—H8B	109.5
N10—Ba2—O7	58.47 (9)	C7—C8—H8B	109.5
O11—Ba2—O8	78.61 (7)	H8A—C8—H8B	108.1
O10—Ba2—O8	143.09 (7)	N2—C9—C10	110.5 (3)
N12—Ba2—O8	77.51 (7)	N2—C9—H9B	109.5
N10—Ba2—O8	58.82 (8)	C10—C9—H9B	109.5
O7—Ba2—O8	117.22 (8)	N2—C9—H9C	109.5
O11—Ba2—O6	72.53 (7)	C10—C9—H9C	109.5
O10—Ba2—O6	65.32 (7)	H9B—C9—H9C	108.1
N12—Ba2—O6	125.57 (8)	O4—C10—C9	109.3 (2)
N10—Ba2—O6	110.74 (8)	O4—C10—H10A	109.8
O7—Ba2—O6	58.40 (8)	C9—C10—H10A	109.8
O8—Ba2—O6	151.00 (7)	O4—C10—H10B	109.8
O11—Ba2—O9	75.86 (7)	C9—C10—H10B	109.8
O10—Ba2—O9	115.52 (7)	H10A—C10—H10B	108.3
N12—Ba2—O9	67.84 (7)	O4—C11—C12	109.7 (3)
N10—Ba2—O9	111.03 (8)	O4—C11—H11A	109.7
O7—Ba2—O9	151.71 (7)	C12—C11—H11A	109.7
O8—Ba2—O9	58.02 (8)	O4—C11—H11B	109.7
O6—Ba2—O9	110.70 (8)	C12—C11—H11B	109.7
O11—Ba2—N11	84.00 (7)	H11A—C11—H11B	108.2
O10—Ba2—N11	71.79 (7)	O1—C12—C11	108.6 (2)
N12—Ba2—N11	81.66 (8)	O1—C12—H12A	110.0
N10—Ba2—N11	151.42 (8)	C11—C12—H12A	110.0
O7—Ba2—N11	117.33 (9)	O1—C12—H12B	110.0
O8—Ba2—N11	115.65 (8)	C11—C12—H12B	110.0
O6—Ba2—N11	58.97 (9)	H12A—C12—H12B	108.3
O9—Ba2—N11	57.70 (9)	N4—C13—Au1	177.1 (3)
O11—Ba2—N9	134.24 (7)	N3—C14—Au1	179.2 (3)
O10—Ba2—N9	66.20 (7)	N5—C15—Au2	176.8 (3)
N12—Ba2—N9	75.11 (7)	N6—C16—Au3	179.2 (3)
N10—Ba2—N9	70.20 (8)	N7—C17—Au3	179.1 (3)
O7—Ba2—N9	67.68 (7)	N8—C18—Au4	176.4 (3)
O8—Ba2—N9	93.98 (7)	N9—C19—Au4	177.2 (3)
O6—Ba2—N9	108.12 (7)	O6—C20—C21	108.0 (3)
O9—Ba2—N9	137.08 (7)	O6—C20—H20A	110.1
N11—Ba2—N9	137.13 (8)	C21—C20—H20A	110.1
C13—Au1—C14	177.12 (14)	O6—C20—H20B	110.1
C15^i^—Au2—C15	179.999 (1)	C21—C20—H20B	110.1
C16—Au3—C17	177.40 (13)	H20A—C20—H20B	108.4
C16—Au3—Au4	87.75 (9)	O7—C21—C20	111.0 (3)
C17—Au3—Au4	94.85 (9)	O7—C21—H21A	109.4
C18—Au4—C19	176.60 (12)	C20—C21—H21A	109.4
C18—Au4—Au3	101.99 (9)	O7—C21—H21B	109.4
C19—Au4—Au3	81.12 (8)	C20—C21—H21B	109.4
C32^ii^—Au5—C32	179.998 (2)	H21A—C21—H21B	108.0
C12—O1—C1	111.8 (2)	O7—C22—C23	110.4 (3)
C12—O1—Ba1	115.33 (16)	O7—C22—H22A	109.6
C1—O1—Ba1	116.57 (18)	C23—C22—H22A	109.6
C5—O2—C4	111.7 (2)	O7—C22—H22B	109.6
C5—O2—Ba1	113.39 (17)	C23—C22—H22B	109.6
C4—O2—Ba1	119.24 (18)	H22A—C22—H22B	108.1
C7—O3—C6	110.9 (2)	N10—C23—C22	111.5 (3)
C7—O3—Ba1	118.93 (17)	N10—C23—H23A	109.3
C6—O3—Ba1	119.53 (17)	C22—C23—H23A	109.3
C11—O4—C10	110.8 (2)	N10—C23—H23B	109.3
C11—O4—Ba1	119.65 (16)	C22—C23—H23B	109.3
C10—O4—Ba1	121.32 (17)	H23A—C23—H23B	108.0
Ba1—O5—H5C	101 (3)	N10—C24—C25	109.8 (3)
Ba1—O5—H5D	136 (3)	N10—C24—H24A	109.7
H5C—O5—H5D	107 (3)	C25—C24—H24A	109.7
C20—O6—C31	111.5 (3)	N10—C24—H24B	109.7
C20—O6—Ba2	111.6 (2)	C25—C24—H24B	109.7
C31—O6—Ba2	114.4 (2)	H24A—C24—H24B	108.2
C22—O7—C21	111.1 (3)	O8—C25—C24	110.8 (3)
C22—O7—Ba2	120.4 (2)	O8—C25—H25A	109.5
C21—O7—Ba2	116.8 (2)	C24—C25—H25A	109.5
C26—O8—C25	111.7 (3)	O8—C25—H25B	109.5
C26—O8—Ba2	120.0 (2)	C24—C25—H25B	109.5
C25—O8—Ba2	117.8 (2)	H25A—C25—H25B	108.1
C28—O9—C27	113.4 (3)	O8—C26—C27	109.3 (3)
C28—O9—Ba2	115.4 (2)	O8—C26—H26A	109.8
C27—O9—Ba2	112.4 (2)	C27—C26—H26A	109.8
Ba2—O10—H10C	120 (2)	O8—C26—H26B	109.8
Ba2—O10—H10D	121 (2)	C27—C26—H26B	109.8
H10C—O10—H10D	106 (3)	H26A—C26—H26B	108.3
Ba2—O11—H11C	121 (2)	O9—C27—C26	110.1 (3)
Ba2—O11—H11D	134 (2)	O9—C27—H27A	109.6
H11C—O11—H11D	104 (3)	C26—C27—H27A	109.6
C3—N1—C2	112.9 (2)	O9—C27—H27B	109.6
C3—N1—Ba1	114.07 (18)	C26—C27—H27B	109.6
C2—N1—Ba1	112.68 (18)	H27A—C27—H27B	108.2
C3—N1—H1	111 (2)	O9—C28—C29	108.8 (3)
C2—N1—H1	105 (2)	O9—C28—H28A	109.9
Ba1—N1—H1	100 (2)	C29—C28—H28A	109.9
C8—N2—C9	112.6 (2)	O9—C28—H28B	109.9
C8—N2—Ba1	107.68 (17)	C29—C28—H28B	109.9
C9—N2—Ba1	109.42 (17)	H28A—C28—H28B	108.3
C8—N2—H2	110 (2)	N11—C29—C28	112.2 (3)
C9—N2—H2	104 (2)	N11—C29—H29A	109.2
Ba1—N2—H2	113 (2)	C28—C29—H29A	109.2
C15—N5—Ba1	158.4 (2)	N11—C29—H29B	109.2
C16—N6—Ba1	137.8 (2)	C28—C29—H29B	109.2
C19—N9—Ba2	149.6 (2)	H29A—C29—H29B	107.9
C24—N10—C23	113.0 (3)	N11—C30—C31	111.3 (3)
C24—N10—Ba2	112.3 (2)	N11—C30—H30A	109.4
C23—N10—Ba2	115.7 (2)	C31—C30—H30A	109.4
C24—N10—H10	100 (3)	N11—C30—H30B	109.4
C23—N10—H10	116 (3)	C31—C30—H30B	109.4
Ba2—N10—H10	98 (3)	H30A—C30—H30B	108.0
C30—N11—C29	111.7 (3)	O6—C31—C30	108.3 (3)
C30—N11—Ba2	115.4 (2)	O6—C31—H31A	110.0
C29—N11—Ba2	118.3 (2)	C30—C31—H31A	110.0
C30—N11—H11	105 (3)	O6—C31—H31B	110.0
C29—N11—H11	110 (3)	C30—C31—H31B	110.0
Ba2—N11—H11	95 (3)	H31A—C31—H31B	108.4
C32—N12—Ba2	153.4 (2)	N12—C32—Au5	178.2 (3)
O1—C1—C2	108.5 (2)		
			
C14—Au1—Au2—C15	53.73 (13)	O3—Ba1—N1—C2	141.49 (19)
C13—Au1—Au2—C15	−127.71 (13)	O2—Ba1—N1—C2	143.1 (2)
C14—Au1—Au2—C15^i^	−126.27 (13)	O4—Ba1—N1—C2	−13.3 (2)
C13—Au1—Au2—C15^i^	52.29 (13)	O1—Ba1—N1—C2	−13.15 (19)
C16—Au3—Au4—C18	−125.59 (12)	N6—Ba1—N1—C2	67.2 (2)
C17—Au3—Au4—C18	54.37 (13)	N5—Ba1—N1—C2	−119.6 (2)
C16—Au3—Au4—C19	55.81 (12)	N2—Ba1—N1—C2	61.5 (3)
C17—Au3—Au4—C19	−124.22 (13)	O5—Ba1—N2—C8	−51.2 (2)
O5—Ba1—O1—C12	−95.75 (19)	O3—Ba1—N2—C8	25.70 (18)
O3—Ba1—O1—C12	115.7 (2)	O2—Ba1—N2—C8	50.2 (2)
O2—Ba1—O1—C12	−176.93 (18)	O4—Ba1—N2—C8	−152.5 (2)
O4—Ba1—O1—C12	25.14 (18)	O1—Ba1—N2—C8	−177.53 (18)
N6—Ba1—O1—C12	120.7 (2)	N6—Ba1—N2—C8	113.4 (2)
N5—Ba1—O1—C12	−29.2 (2)	N5—Ba1—N2—C8	−59.96 (19)
N2—Ba1—O1—C12	50.4 (2)	N1—Ba1—N2—C8	119.2 (2)
N1—Ba1—O1—C12	−154.7 (2)	O5—Ba1—N2—C9	71.4 (2)
O5—Ba1—O1—C1	38.4 (2)	O3—Ba1—N2—C9	148.3 (2)
O3—Ba1—O1—C1	−110.1 (2)	O2—Ba1—N2—C9	172.83 (17)
O2—Ba1—O1—C1	−42.8 (2)	O4—Ba1—N2—C9	−29.84 (17)
O4—Ba1—O1—C1	159.3 (2)	O1—Ba1—N2—C9	−54.9 (2)
N6—Ba1—O1—C1	−105.1 (2)	N6—Ba1—N2—C9	−123.9 (2)
N5—Ba1—O1—C1	104.9 (2)	N5—Ba1—N2—C9	62.67 (19)
N2—Ba1—O1—C1	−175.44 (19)	N1—Ba1—N2—C9	−118.2 (2)
N1—Ba1—O1—C1	−20.59 (19)	O5—Ba1—N5—C15	92.1 (6)
O5—Ba1—O2—C5	84.9 (2)	O3—Ba1—N5—C15	−157.5 (6)
O3—Ba1—O2—C5	−27.02 (19)	O2—Ba1—N5—C15	156.8 (6)
O4—Ba1—O2—C5	−119.6 (2)	O4—Ba1—N5—C15	−34.6 (6)
O1—Ba1—O2—C5	176.92 (19)	O1—Ba1—N5—C15	9.6 (7)
N6—Ba1—O2—C5	−118.5 (2)	N6—Ba1—N5—C15	−106.4 (6)
N5—Ba1—O2—C5	27.1 (2)	N2—Ba1—N5—C15	−94.4 (6)
N2—Ba1—O2—C5	−51.9 (2)	N1—Ba1—N5—C15	86.0 (6)
N1—Ba1—O2—C5	154.6 (2)	O5—Ba1—N6—C16	4.4 (4)
O5—Ba1—O2—C4	−49.8 (2)	O3—Ba1—N6—C16	−96.5 (3)
O3—Ba1—O2—C4	−161.8 (2)	O2—Ba1—N6—C16	−36.4 (3)
O4—Ba1—O2—C4	105.7 (2)	O4—Ba1—N6—C16	143.2 (3)
O1—Ba1—O2—C4	42.2 (2)	O1—Ba1—N6—C16	85.4 (3)
N6—Ba1—O2—C4	106.7 (2)	N5—Ba1—N6—C16	−146.4 (3)
N5—Ba1—O2—C4	−107.6 (2)	N2—Ba1—N6—C16	−157.9 (4)
N2—Ba1—O2—C4	173.4 (2)	N1—Ba1—N6—C16	24.5 (3)
N1—Ba1—O2—C4	19.9 (2)	O11—Ba2—N9—C19	38.5 (5)
O5—Ba1—O3—C7	143.6 (2)	O10—Ba2—N9—C19	−95.8 (5)
O2—Ba1—O3—C7	−147.1 (2)	N12—Ba2—N9—C19	−167.6 (5)
O4—Ba1—O3—C7	8.8 (2)	N10—Ba2—N9—C19	61.7 (5)
O1—Ba1—O3—C7	−68.5 (3)	O7—Ba2—N9—C19	−1.4 (5)
N6—Ba1—O3—C7	−73.4 (2)	O8—Ba2—N9—C19	116.4 (5)
N5—Ba1—O3—C7	81.4 (2)	O6—Ba2—N9—C19	−44.5 (5)
N2—Ba1—O3—C7	7.0 (2)	O9—Ba2—N9—C19	161.6 (4)
N1—Ba1—O3—C7	−145.5 (2)	N11—Ba2—N9—C19	−108.1 (5)
O5—Ba1—O3—C6	−75.0 (2)	O11—Ba2—N10—C24	−120.0 (3)
O2—Ba1—O3—C6	−5.7 (2)	O10—Ba2—N10—C24	105.5 (3)
O4—Ba1—O3—C6	150.1 (2)	N12—Ba2—N10—C24	19.6 (3)
O1—Ba1—O3—C6	72.8 (3)	O7—Ba2—N10—C24	153.1 (3)
N6—Ba1—O3—C6	67.9 (2)	O8—Ba2—N10—C24	−29.9 (2)
N5—Ba1—O3—C6	−137.2 (2)	O6—Ba2—N10—C24	−179.7 (2)
N2—Ba1—O3—C6	148.3 (2)	O9—Ba2—N10—C24	−56.3 (3)
N1—Ba1—O3—C6	−4.1 (2)	N11—Ba2—N10—C24	−116.9 (3)
O5—Ba1—O4—C11	85.4 (2)	N9—Ba2—N10—C24	77.8 (3)
O3—Ba1—O4—C11	−147.1 (2)	O11—Ba2—N10—C23	108.2 (3)
O2—Ba1—O4—C11	−66.7 (3)	O10—Ba2—N10—C23	−26.3 (3)
O1—Ba1—O4—C11	7.2 (2)	N12—Ba2—N10—C23	−112.1 (2)
N6—Ba1—O4—C11	−67.8 (2)	O7—Ba2—N10—C23	21.3 (2)
N5—Ba1—O4—C11	143.2 (2)	O8—Ba2—N10—C23	−161.7 (3)
N2—Ba1—O4—C11	−145.2 (2)	O6—Ba2—N10—C23	48.5 (3)
N1—Ba1—O4—C11	7.3 (2)	O9—Ba2—N10—C23	171.9 (2)
O5—Ba1—O4—C10	−129.2 (2)	N11—Ba2—N10—C23	111.3 (3)
O3—Ba1—O4—C10	−1.6 (2)	N9—Ba2—N10—C23	−54.0 (2)
O2—Ba1—O4—C10	78.7 (3)	O11—Ba2—N11—C30	−63.8 (2)
O1—Ba1—O4—C10	152.7 (2)	O10—Ba2—N11—C30	81.0 (2)
N6—Ba1—O4—C10	77.7 (2)	N12—Ba2—N11—C30	150.2 (3)
N5—Ba1—O4—C10	−71.4 (2)	N10—Ba2—N11—C30	−66.7 (3)
N2—Ba1—O4—C10	0.2 (2)	O7—Ba2—N11—C30	6.9 (3)
N1—Ba1—O4—C10	152.8 (2)	O8—Ba2—N11—C30	−138.1 (2)
O11—Ba2—O6—C20	−114.7 (2)	O6—Ba2—N11—C30	9.4 (2)
O10—Ba2—O6—C20	69.0 (2)	O9—Ba2—N11—C30	−140.8 (3)
N12—Ba2—O6—C20	101.5 (2)	N9—Ba2—N11—C30	92.8 (3)
N10—Ba2—O6—C20	−58.0 (3)	O11—Ba2—N11—C29	72.4 (3)
O7—Ba2—O6—C20	−30.8 (2)	O10—Ba2—N11—C29	−142.8 (3)
O8—Ba2—O6—C20	−120.7 (3)	N12—Ba2—N11—C29	−73.6 (3)
O9—Ba2—O6—C20	178.4 (2)	N10—Ba2—N11—C29	69.5 (3)
N11—Ba2—O6—C20	151.8 (3)	O7—Ba2—N11—C29	143.1 (2)
N9—Ba2—O6—C20	17.1 (3)	O8—Ba2—N11—C29	−1.9 (3)
O11—Ba2—O6—C31	117.5 (2)	O6—Ba2—N11—C29	145.6 (3)
O10—Ba2—O6—C31	−58.9 (2)	O9—Ba2—N11—C29	−4.6 (2)
N12—Ba2—O6—C31	−26.4 (3)	N9—Ba2—N11—C29	−131.0 (2)
N10—Ba2—O6—C31	174.2 (2)	O11—Ba2—N12—C32	−83.9 (5)
O7—Ba2—O6—C31	−158.6 (2)	O10—Ba2—N12—C32	57.8 (5)
O8—Ba2—O6—C31	111.5 (3)	N10—Ba2—N12—C32	−176.4 (5)
O9—Ba2—O6—C31	50.6 (2)	O7—Ba2—N12—C32	107.6 (5)
N11—Ba2—O6—C31	24.0 (2)	O8—Ba2—N12—C32	−134.6 (5)
N9—Ba2—O6—C31	−110.7 (2)	O6—Ba2—N12—C32	26.0 (5)
O11—Ba2—O7—C22	−63.9 (2)	O9—Ba2—N12—C32	−74.3 (5)
O10—Ba2—O7—C22	154.2 (2)	N11—Ba2—N12—C32	−15.8 (5)
N12—Ba2—O7—C22	109.1 (2)	N9—Ba2—N12—C32	127.8 (5)
N10—Ba2—O7—C22	8.1 (2)	C12—O1—C1—C2	−172.1 (3)
O8—Ba2—O7—C22	5.2 (3)	Ba1—O1—C1—C2	52.2 (3)
O6—Ba2—O7—C22	−141.8 (3)	C3—N1—C2—C1	176.1 (3)
O9—Ba2—O7—C22	−67.3 (3)	Ba1—N1—C2—C1	45.0 (3)
N11—Ba2—O7—C22	−139.3 (2)	O1—C1—C2—N1	−65.0 (3)
N9—Ba2—O7—C22	87.9 (2)	C2—N1—C3—C4	−172.5 (3)
O11—Ba2—O7—C21	76.0 (2)	Ba1—N1—C3—C4	−42.2 (3)
O10—Ba2—O7—C21	−66.0 (2)	C5—O2—C4—C3	175.2 (3)
N12—Ba2—O7—C21	−111.1 (2)	Ba1—O2—C4—C3	−49.4 (3)
N10—Ba2—O7—C21	147.9 (3)	N1—C3—C4—O2	59.9 (3)
O8—Ba2—O7—C21	145.0 (2)	C4—O2—C5—C6	−165.0 (3)
O6—Ba2—O7—C21	−2.0 (2)	Ba1—O2—C5—C6	56.8 (3)
O9—Ba2—O7—C21	72.5 (3)	C7—O3—C6—C5	179.6 (3)
N11—Ba2—O7—C21	0.5 (3)	Ba1—O3—C6—C5	35.4 (3)
N9—Ba2—O7—C21	−132.3 (3)	O2—C5—C6—O3	−60.2 (3)
O11—Ba2—O8—C26	−72.1 (2)	C6—O3—C7—C8	177.4 (3)
O10—Ba2—O8—C26	99.1 (3)	Ba1—O3—C7—C8	−38.2 (3)
N12—Ba2—O8—C26	79.8 (2)	C9—N2—C8—C7	−178.6 (3)
N10—Ba2—O8—C26	−142.5 (3)	Ba1—N2—C8—C7	−57.9 (3)
O7—Ba2—O8—C26	−139.5 (2)	O3—C7—C8—N2	65.8 (3)
O6—Ba2—O8—C26	−66.2 (3)	C8—N2—C9—C10	179.1 (3)
O9—Ba2—O8—C26	8.3 (2)	Ba1—N2—C9—C10	59.4 (3)
N11—Ba2—O8—C26	5.5 (3)	C11—O4—C10—C9	176.9 (3)
N9—Ba2—O8—C26	153.6 (2)	Ba1—O4—C10—C9	28.7 (3)
O11—Ba2—O8—C25	69.8 (2)	N2—C9—C10—O4	−59.1 (3)
O10—Ba2—O8—C25	−119.0 (2)	C10—O4—C11—C12	174.6 (3)
N12—Ba2—O8—C25	−138.3 (2)	Ba1—O4—C11—C12	−36.6 (3)
N10—Ba2—O8—C25	−0.6 (2)	C1—O1—C12—C11	169.5 (3)
O7—Ba2—O8—C25	2.4 (3)	Ba1—O1—C12—C11	−54.2 (3)
O6—Ba2—O8—C25	75.7 (3)	O4—C11—C12—O1	58.7 (3)
O9—Ba2—O8—C25	150.2 (3)	C31—O6—C20—C21	−169.0 (3)
N11—Ba2—O8—C25	147.4 (2)	Ba2—O6—C20—C21	61.6 (3)
N9—Ba2—O8—C25	−64.5 (2)	C22—O7—C21—C20	176.5 (3)
O11—Ba2—O9—C28	−118.5 (2)	Ba2—O7—C21—C20	33.1 (4)
O10—Ba2—O9—C28	18.0 (3)	O6—C20—C21—O7	−63.5 (4)
N12—Ba2—O9—C28	67.5 (2)	C21—O7—C22—C23	−177.0 (3)
N10—Ba2—O9—C28	−177.1 (2)	Ba2—O7—C22—C23	−35.1 (4)
O7—Ba2—O9—C28	−115.0 (3)	C24—N10—C23—C22	179.7 (3)
O8—Ba2—O9—C28	156.3 (3)	Ba2—N10—C23—C22	−48.9 (3)
O6—Ba2—O9—C28	−53.7 (2)	O7—C22—C23—N10	54.6 (4)
N11—Ba2—O9—C28	−26.7 (2)	C23—N10—C24—C25	−167.2 (3)
N9—Ba2—O9—C28	99.7 (2)	Ba2—N10—C24—C25	59.7 (3)
O11—Ba2—O9—C27	109.4 (2)	C26—O8—C25—C24	174.7 (3)
O10—Ba2—O9—C27	−114.1 (2)	Ba2—O8—C25—C24	29.8 (4)
N12—Ba2—O9—C27	−64.6 (2)	N10—C24—C25—O8	−59.4 (4)
N10—Ba2—O9—C27	50.8 (2)	C25—O8—C26—C27	177.6 (3)
O7—Ba2—O9—C27	112.9 (3)	Ba2—O8—C26—C27	−38.4 (4)
O8—Ba2—O9—C27	24.1 (2)	C28—O9—C27—C26	172.1 (3)
O6—Ba2—O9—C27	174.2 (2)	Ba2—O9—C27—C26	−54.8 (3)
N11—Ba2—O9—C27	−158.8 (3)	O8—C26—C27—O9	61.8 (4)
N9—Ba2—O9—C27	−32.4 (3)	C27—O9—C28—C29	−173.7 (3)
O5—Ba1—N1—C3	103.9 (2)	Ba2—O9—C28—C29	54.6 (3)
O3—Ba1—N1—C3	11.0 (2)	C30—N11—C29—C28	171.3 (3)
O2—Ba1—N1—C3	12.63 (19)	Ba2—N11—C29—C28	33.6 (4)
O4—Ba1—N1—C3	−143.73 (19)	O9—C28—C29—N11	−58.0 (4)
O1—Ba1—N1—C3	−143.6 (2)	C29—N11—C30—C31	−179.9 (3)
N6—Ba1—N1—C3	−63.3 (2)	Ba2—N11—C30—C31	−40.9 (4)
N5—Ba1—N1—C3	109.9 (2)	C20—O6—C31—C30	177.6 (3)
N2—Ba1—N1—C3	−69.0 (3)	Ba2—O6—C31—C30	−54.6 (4)
O5—Ba1—N1—C2	−125.7 (2)	N11—C30—C31—O6	63.8 (4)

Symmetry codes: (i) −*x*+2, −*y*, −*z*+2; (ii) −*x*+2, −*y*+1, −*z*+2.

### Hydrogen-bond geometry (Å, °)

**Table:**

*D*—H···*A*	*D*—H	H···*A*	*D*···*A*	*D*—H···*A*
O5—H5C···N4^i^	0.84 (3)	2.19 (2)	2.997 (4)	161 (4)
O5—H5D···N3^iii^	0.84 (3)	1.98 (3)	2.804 (4)	168 (3)
O10—H10C···N8^iv^	0.83 (4)	2.09 (3)	2.916 (3)	170 (3)
O10—H10D···N6	0.84 (4)	2.35 (2)	3.132 (3)	156 (3)
O11—H11C···N4^ii^	0.84 (4)	2.01 (3)	2.845 (4)	177 (3)
O11—H11D···N7^v^	0.84 (3)	2.09 (3)	2.920 (4)	176 (4)

Symmetry codes: (i) −*x*+2, −*y*, −*z*+2; (iii) −*x*+1, −*y*, −*z*+2; (iv) *x*+1, *y*, *z*; (ii) −*x*+2, −*y*+1, −*z*+2; (v) −*x*+1, −*y*+2, −*z*+1.
